# Utilization of physical therapy services in Germany

**DOI:** 10.17886/RKI-GBE-2017-129

**Published:** 2017-12-13

**Authors:** Alexander Rommel, Prütz Franziska

**Affiliations:** Robert Koch Institute, Department of Epidemiology and Health Monitoring, Berlin

**Keywords:** PHYSICAL THERAPY, UTILIZATION, HEALTH CARE, HEALTH MONITORING, GERMANY

## Abstract

Physical therapy plays an important role in health care and is listed as a therapy in many clinical guidelines. Claims data gathered by statutory health insurers demonstrate that in Germany physical therapy accounts for more than 70% of the reimbursable costs of all non-medical treatments comprising physical therapy, logopaedics, occupational therapy and podiatry. In monetary terms, this equates to about EUR 4.4 billion annually. According to self-reports of the respondents of GEDA 2014/2015-EHIS on their utilization of physical therapy services 25.5% of women and 17.7% of men used physical therapy services within the 12 months that preceded the interview. This rate increases significantly with age, reaching its peak among the 50-to-59 year age group, at 30.7% among women and 20.1% among men. People with a high level of education and those with private health insurance use physical therapy services more frequently. Moreover, people living in the eastern part of Germany use physical therapy services more often than those living in the western federal states.

## Introduction

Physical therapy includes active exercise-based forms of therapy (such as kinesitherapy and therapeutic exercise) and passive forms of treatment such as heat treatment and massage. Both forms of treatment are aimed at promoting, maintaining and restoring a patient’s physical function and movement as part of the prevention and treatment of disease. Another important field in which physical therapy is applied is the alleviation of symptoms during palliative care.

In accordance with the fifth book of the German Social Code (SGB V), people with statutory health insurance have a right to outpatient non-medical treatment within the statutory health care framework. Alongside physical therapy, this right encompasses occupational therapy, logopaedics and podiatry (in Germany summarised under the term Heilmittel). People with private insurance are also entitled to physical therapy services to varying degrees. In addition, physical therapy is provided during inpatient care in hospitals and as part of medical rehabilitation in accordance with the eleventh book of the German Social Code (SGB XI).

The Federal Joint Committee regulates the provision of physical therapy, logopaedics, occupational therapy and podiatry to people with statutory health insurance in the outpatient field in detail through a certain guideline (Heilmittelrichtlinie) and via a catalogue of reimbursable therapies (Heilmittelkatalog). Accordingly, physical therapy is carried out on medical prescription and the costs are covered by statutory health insurers, apart from fixed co-payments. To a limited extent, alternative practitioners also provide physical therapy services that have not been medically prescribed and therefore have to be paid for privately (out of pocket). Claims data gathered by statutory health insurers demonstrate that physical therapy accounts for more than 70% of the costs these insurers incur for the coverage of all non-medical treatments comprising physical therapy, logopaedics, occupational therapy and podiatry. In 2015, this corresponded to EUR 4.4 billion [1].

**GEDA 2014/2015-EHIS**

**Data holder:** Robert Koch Institute

**Aims:** To provide reliable information about the population’s health status, health-related behaviour and health care in Germany, with the possibility of a European comparison

**Method:** Questionnaires completed on paper or online

**Population:** People aged 18 years and above with permanent residency in Germany

**Sampling:** Registry office sample; randomly selected individuals from 301 communities in Germany were invited to participate

**Participants:** 24,016 people (13,144 women; 10,872 men)

**Response rate:** 26.9%

**Study period:** November 2014 - July 2015

**Data protection:** This study was undertaken in strict accordance with the data protection regulations set out in the German Federal Data Protection Act and was approved by the German Federal Commissioner for Data Protection and Freedom of Information. Participation in the study was voluntary. The participants were fully informed about the study’s aims and content, and about data protection. All participants provided written informed consent.

More information in German is available at www.geda-studie.de

Although physical therapy includes a wide range of therapeutic approaches, active exercise-based forms of therapy are used most frequently. Thus, almost two thirds of patients who are prescribed physical therapy receive active exercise therapy [1]. Musculoskeletal disorders are among the most common conditions that indicate a need for physical therapy. According to the statutory health insurer AOK, in 2015, a diagnosis of back pain (ICD-10: M54) was the reason for treatment in around one third of cases of patients receiving physical therapy. In addition, nearly 10% of physical therapy patients suffered from arthritis with different localisations (ICD-10: M16, M17, M19). Finally, other musculoskeletal disorders as well as neurological disorders (such as strokes) are further important reasons that point to a need for physical therapy [1].

Physical therapy procedures have found their way into a variety of clinical guidelines on the treatment of conditions far beyond those that have been mentioned. These also include clinical guidelines on the treatment of diseases such as malignant neoplasms and pulmonary diseases [2, 3]. Physical therapy therefore constitutes an important and integral aspect of health care provision in Germany. Data on the costs and the services that are provided continue to be largely gathered from claims data held by statutory health insurers [1, 4-7]. However, the data collected by the Robert Koch Institute’s health monitoring system allow for population-wide analyses of the utilization of physical therapy and help to identify related social determinants [8]. Current data on the utilization of physical therapy are available from the German Health Update (GEDA 2014/2015-EHIS) study.

## Indicator

In the GEDA 2014/2015-EHIS study, data on the utilization of physical therapy services were collected using a questionnaire filled out by respondents either on paper or online. In the case of physical therapy, respondents were asked: ‘Have you visited a physical therapist in the last 12 months for consultation, examination or treatment?’ The respondents were able to answer with ‘yes’ or ‘no’. This question forms part of the European Health Interview Survey (EHIS), which has been adopted as an obligatory research programme by all EU member states in accordance with EU regulations [9, 10].

The following analyses are based on information gathered from 23,917 participants aged 18 years and above (13,095 women; 10,822 men) with valid data on physical therapy utilization. The calculations were carried out using a weighting factor that corrected the sample for deviations from the German population structure (as of 31 December 2014) in terms of gender, age, district type and level of education. The district type reflects the degree of urbanisation and accounts for the regional distribution in Germany. The International Standard Classification of Education (ISCED) was used to classify the information provided by the participants on their level of education [11].

Differences between these groups are interpreted as statistically significant if the respective confidence intervals do not overlap.

A detailed description of the methodology used for GEDA 2014/2015-EHIS can be found in Lange et al. 2017 [12] as well as in the article German Health Update: New data for Germany and Europe in issue 1/2017 of the Journal of Health Monitoring.

## Results and discussion

More than one fifth of the German population (21.7%) reported having used physical therapy services within the 12 months that preceded the interview. This rate is markedly higher among women (25.5%) than men (17.7%). Furthermore, these figures are somewhat higher than the corresponding indicators gained from claims data provided by statutory health insurers [1, 13]. This is due to the fact that GEDA 2014/2015-EHIS also collected data from privately insured people who usually use health care services more often [14]. In addition, the data gained from the questionnaires may also include a limited amount of physical therapy treatments that were paid for by the patients themselves and that were conducted without medical prescription; others may have been provided in hospital, or as part of a programme of medical rehabilitation.

**Figure 1:**
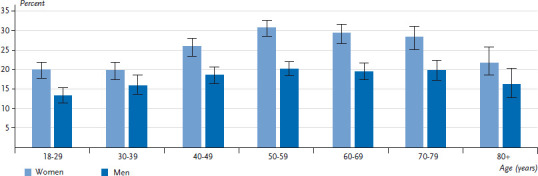
Utilization of physical therapy services according to gender and age (n=13,095 women; n=10,822 men) Source: GEDA 2014/2015-EHIS

The utilization of physical therapy increases significantly with age, reaching its peak among the 50-to-59 year age group, at 30.7% among women and 20.1% among men (Figure 1). The difference between women and men is particularly pronounced in middle age. A decline in the utilization of physical therapy is observed among the elderly (Figure 1) but it is not statistically significant. These socio-demographic differences are consistent with findings that show a more frequent utilization of health services in general among women and of physical therapy in particular [1, 7, 8, 13, 14]. Further analyses have confirmed the higher levels of utilization with increasing age, and the fact that utilization then diminishes among the elderly [7, 8].

In addition to age and gender, significant differences also exist with regard to education (Table 1). The proportion of people with a high level of education using physical therapy is higher than among people with lower levels of education. With regard to the utilization of medical services in general, it is evident that people with better social positions tend to use more specialised and preventive services more often [14-16]. The utilization of physical therapy services also follows this pattern. The differences between educational groups are also confirmed by studies that have statistically controlled for medical need (morbidity) [8, 17]. The differences in utilization, therefore, cannot solely be explained by an unequal distribution of the burden of disease between groups with varying degrees of education.

People with private insurance have a higher rate of physical therapy utilization than people with statutory health insurance (Table 1). Here, too, the differences continue to be present even after medical need has been statistically controlled for [8]. One explanation may be that people with private insurance generally have higher incomes and higher levels of education than people with statutory health insurance. In addition, some alternative treatments such as osteopathy, which are also provided by physical therapists, are only reimbursed by statutory health insurers in exceptional cases. In contrast, private health insurers are more likely to cover the costs of these procedures, which also leads to a higher utilization of physical therapy among people with this form of insurance. In addition, private health insurers offer schemes that absolve their policy holders of any co-payments. In contrast, in accordance with SGB V, people with statutory insurance are required to pay EUR 10 per prescription and to cover 10% of the costs of treatment themselves; this places a financial hurdle on the use of physical therapy by patients with statutory health insurance.

**Figure 2:**
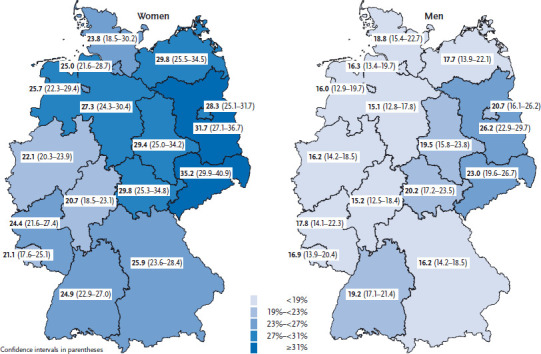
Utilization of physical therapy services according to gender and federal state (n=13,095 women; n=10,822 men) Source: GEDA 2014/2015-EHIS

Finally, the utilization of physical therapy services also differs between regions (Figure 2), with a more than 10-percentage-point difference existing between the highest and lowest levels of utilization throughout Germany. In Saxony 29.2% of the population used physical therapy services in the 12 months that preceded the interview; in Hesse, the same can be said of just 18.0% of the population. In general, physical therapy is used more frequently in the eastern part of Germany and Berlin than in the western federal states. These differences are more pronounced among women than men (Figure 2). The regional distribution of the utilization of physical therapy largely corresponds with claims data held by statutory health insurers [6]. One possible reason behind these differences relates to the existence of regional differences in doctors’ approaches to prescribing physical therapy: according to the statutory health insurer Barmer GEK, the proportion of insured people who are actually prescribed physical therapy by their doctor in case of medical need is significantly higher in the eastern German federal states than in the western part (e.g. Saxony 57.0% versus Hesse 38.1%) [7]. Further studies are needed to determine the extent to which regional differences in the burden of disease are, in turn, responsible for the different levels of utilization of physical therapy services.

Physical therapy is one of the most frequently used health care services in Germany. Its rate of utilization depends not only on medical but also on socio-demographic and health care-related factors (see, for example, [15]). However, in order to ensure that all sections of the population benefit equally from health care services, it is essential that the impact of these factors is minimised. Research should seek to identify the reasons behind the differences in care provision and consider them when making practical recommendations. In this respect, more indication-specific research could be useful. This would also help clarify the non-medical factors influencing utilization and to develop recommendations that have more relevance to day-to-day care.

The data gathered by the Health Monitoring System conducted at the Robert Koch Institute can contribute to the analysis of developments in health care by revealing basic associations of this kind and identifying the need for more specific research and action. Together with other contributions in this issue (Fact sheets on the utilization of outpatient medical care, use of hospital treatment, administration of medically prescribed medicines and self-medication, focus on the utilization of psychotherapeutic and psychiatric treatment), this Fact sheet provides an overview of essential aspects of the utilization of health care provision by adults in Germany.

## Key statements

- 26% of women and 18% of men use physical therapy services within 12 months.
- Physical therapy is most frequently used by people aged between 50 and 59 years.
- People with a high level of education and those with private health insurance use physical therapy more frequently.
- People in the eastern part of Germany visit physical therapists more frequently than people in the western federal states.

## Figures and Tables

**Table 1:** Utilization of physical therapy services according to gender, educational level and insurance status (n=13,095 women; n=10,822 men) Source: GEDA 2014/2015-EHIS

Women	%	(95% CI)	Men	%	(95% CI)
**Women (total)**	**25.5**	**(24.5-26.5)**	**Men (total)**	**17.7**	**(16.8-18.6)**
**Education**			**Education**		
Low education	23.5	(21.5-25.5)	Low education	15.1	(13.2-17.3)
Medium education	25.7	(24.5-26.9)	Medium education	17.7	(16.4-18.9)
High education	27.6	(25.9-29.4)	High education	19.2	(17.8-20.7)
**Insurance status**			**Insurance status**		
Statutory health insurance	24.5	(23.5-25.6)	Statutory health insurance	16.8	(15.8-17.7)
Private health insurance/allowance for civil servants	30.4	(28.0-33.3)	Private health insurance/allowance for civil servants	20.3	(18.5-22.3)
**Total (women and men)**	**21.7**	**(20.9-22.4)**	**Total (women and men)**	**21.7**	**(20.9-22.4)**

CI=confidence interval
